# A new framework for assessment of park management in smart cities: a study based on social media data and deep learning

**DOI:** 10.1038/s41598-024-53345-0

**Published:** 2024-02-13

**Authors:** Sijia Liu, Chuandong Tan, Feiyang Deng, Wei Zhang, Xuefei Wu

**Affiliations:** 1 College of Horticulture and Forestry, Huazhong Agricultural University, Wuhan, 430070 China; 2 College of Urban Design, Wuhan University, Wuhan, 430072 China

**Keywords:** Environmental impact, Sustainability, Information technology, Software

## Abstract

Urban park management assessment is critical to park operation and service quality. Traditional assessment methods cannot comprehensively assess park use and environmental conditions. Besides, although social media and big data have shown significant advantages in understanding public behavior or preference and park features or values, there has been little relevant research on park management assessment. This study proposes a deep learning-based framework for assessing urban park intelligent management from macro to micro levels with comment data from social media. By taking seven parks in Wuhan City as the objects, this study quantitatively assesses their overall state and performance in facilities, safety, environment, activities, and services, and reveals their main problems in management. The results demonstrate the impacts of various factors, including park type, season, and specific events such as remodeling and refurbishment, on visitor satisfaction and the characteristics of individual parks and their management. Compared with traditional methods, this framework enables real-time intelligent assessment of park management, which can accurately reflect park use and visitor feedback, and improve park service quality and management efficiency. Overall, this study provides important reference for intelligent park management assessment based on big data and artificial intelligence, which can facilitate the future development of smart cities.

## Introduction

As an important part of the urban public environment, urban parks not only provide space for the leisure, recreation, and fitness of urban residents, but also increase the green space and improve the ecological environment of cities^1,2^. Urban park management is crucial for ensuring the proper functioning of parks and improving the quality of park services. Assessment of park management and services can identify the problems in park management and propose improvement measures to support effective urban park management^3^.

Traditionally, assessment of park management is conducted mainly through questionnaire surveys, on-site observation, and expert assessment^3–6^. For example, Lin and Liu^6^ carried out a structured questionnaire survey on visitors to the National Xinhua Metropolitan Forest Park in Taiwan and obtained 181 survey samples to assess the management performance of the park. Hsiao^5^ assessed park management and financial effectiveness after introduction of Installation-Management Permission for park lawn based on park user characteristics obtained from questionnaires, observational surveys of park users, and analysis of data from tracking records. Mohamed Ahmed Said^4^ developed a new tool for assessing parks through literature review, a field survey of Khartoum Park's facilities, services, landscapes, green spaces, playgrounds and water features, and had seven professional architects to assess the open space characteristics of six parks. However, the existing park management assessment methodologies generally have some major limitations. User surveys can only collect information from those completing the questionnaire, and therefore may not provide a full picture of park use and environmental quality, and are also time- and labor-intensive^7^. On-site observation allows direct observation of the conditions of park facilities and environment; however, it heavily relies on subjective perceptions and may generate biased assessment results. In addition, on-site observation can only reflect the state of the park over a short period of time and cannot represent the long-term dynamic changes^8,9^. Expert assessment, on the other hand, usually requires relevant expertise and experience, but different experts may have different views and assessment criteria^10^, and therefore the assessment may be influenced by subjective factors such as personal preference and experience^11,12^.

With the proliferation of social media and smart devices, new data sources are emerging and the use of social media data for park research has become an effective and practical approach^7^. Data such as photos, comments, and check-ins posted by users on social media platforms can provide a comprehensive picture of users' perceptions, evaluations, use, and needs of parks^13^. These data are real-time, large-volume, and multidimensional^14^, which can provide park management with more valuable information to further improve the quality of park services and management^8^. A review of previous studies has demonstrated that analysis of social media data on urban parks has been mainly focused on the behavior and preference of park users, such as the number of users, frequency of use, behavioral trajectories and preferences^9,15,16^. Some studies have also explored the impact of parks on visitors' mental health, as well as other aspects such as park landscape design, spatial use, and cultural values^17–20^. For example, Mou et al.^7^ used social media data to analyze the behavior of visitors to urban parks in Beijing and explored the relationship between flowers and park visits. Plunz et al.^21^ used social media data to explore the relationship between urban green spaces and resident well-being. Schwartz et al.^18^ employed the Twitter data to examine the relationship between resident mental health and biodiversity exposure, particularly in urban areas with increasing concern about the rising prevalence of mood disorders. Huang et al.^8^ used user-generated content (UGC) to explore the urban-scale Chicago park management issues, and the role of social media as a tool to communicate visitor feedback to city parks and recreation department. Zhang et al.^19^ used visitor online comments to examine the impact of cultural aspects of Chinese theme parks on visitor sentiment, providing implications for the development of marketing and operational strategies. While social media data have shown significant advantages in understanding public behavior and preference and revealing park features and values, there is still a lack of systematic research on park management assessment with such data, particularly in terms of deeper insights into visitor sentiment feedback and management issues. Therefore, it is necessary to make more effective use of social media data to obtain more comprehensive information and provide better feedback and solutions for park management.

This study proposes a new framework for intelligent assessment of park management by analyzing the user comments on social media in real time to extract problems in park management at the macro, meso, and micro levels. Specifically, we used deep learning techniques for multi-categorization and sentiment analysis on the online comments of users, classified the comments into five categories, including facilities, safety, environment, activities, and services, and performed quantitative scoring of visitor sentiment. Moreover, the main problems in park management were mined through feature word extraction. The framework can enable real-time intelligent assessment of park management to accurately reflect park use and visitor feedback, which can not only help improve park management efficiency, but also enable park managers to understand and solve specific problems in a timely manner. Overall, this study provides a new framework of park management assessment based on big data and artificial intelligence (AI), offering new possibilities for park management in smart cities, which is of great theoretical and practical significance in promoting modern and intelligent park governance and improving the life quality of urban residents.

## Materials and methods

### A framework for intelligent assessment of park management based on deep learning text mining

**Figure 1:**
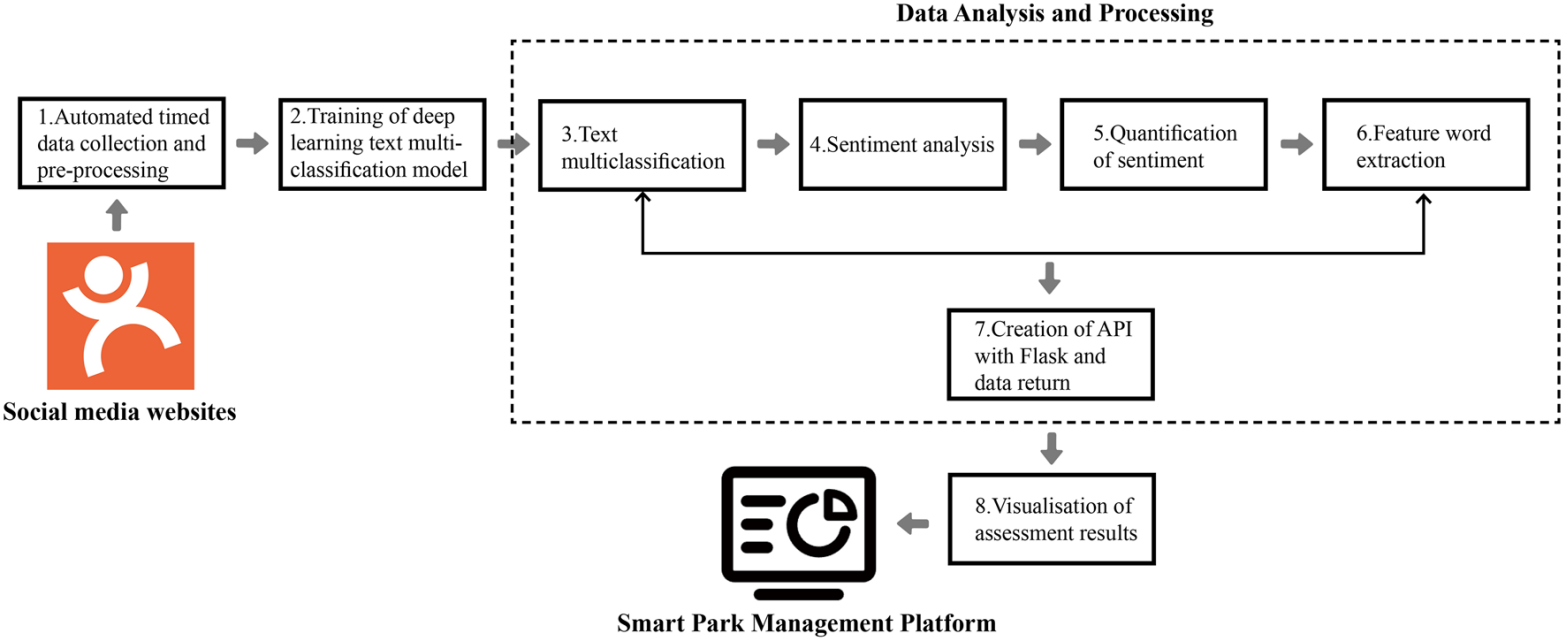
A framework for intelligent assessment of park management based on deep learning text mining.

This work builds a new framework for intelligent assessment of park management based on deep learning text mining at the macro, meso, and micro levels. The framework takes a data-driven approach to comprehensively assess the performance of parks in various aspects of management by analyzing the users' park-related comments on social media and presenting the results through dynamic visualization. Figure 1 depicts the main components of this framework, mainly including (1) a data collection and pre-processing module, which is responsible for automatic extraction of social media data using scripts as well as cleaning of the data to ensure the accuracy and reliability of the subsequent analysis; (2) a training module of deep learning multi-text classification model, which can train the model to classify the visitor comments from various perspectives such as the environment and facilities; (3) a data analysis and processing module, which employs techniques such as sentiment analysis and feature word extraction to mine the valuable information in the comments and quantify sentiment scores; and (4) a module for visualization of the assessment results, which graphically displays the assessment results to visually present the indicators and problems of park management.

### Study area

As a transportation hub and a center of science, education, and culture in central China, Wuhan City is rich in natural resources with many urban parks^22^. Urban parks in Wuhan vary greatly in terms of management mode, facility completeness, and visitor demand, providing a diverse research population and a rich source of data for this study. In addition, Wuhan has made remarkable achievements in urban greening and ecological construction in recent years^23^, which is highly relevant to the assessment of its park management.

Seven representative urban parks in Wuhan were selected for this study, including Yellow Crane Tower Park, Zhongshan Park, Jiefang Park, Wuhan Zoo, Yuehu Park, Guishan Park, and Shahu Park. These seven parks are highly influential in Wuhan. They are all administrated by Wuhan Municipal Bureau of Landscape and Forestry, but differ in terms of type, scale, function, and influence (Table 1)^24^. By taking these seven parks as the research subjects, this study may provide targeted recommendations for the management of urban parks in Wuhan.

**Table 1:** Basic information of the seven studied parks.

Park name	Geographical coordinates	Park type	Park area (hectares)	Ranking on Dianping (popularity first)
Yellow Crane Tower Park	30°32′35.9″N, 114°18′17.8″E (WGS84)	History and Culture (The Yellow Crane Tower Park is a city park with the Yellow Crane Tower as the core attraction, integrating culture, history, gardens and tourism, and is also one of the most famous tourist attractions in Wuhan)	40.3	1 (All categories)
Zhongshan Park	30°35′06.4″N, 114°16′22.0″E (WGS84)	Comprehensive (With the theme of the revolutionary forefather Dr. Sun Yat-sen, Zhongshan Park has a variety of functions such as cultural experience and sports, and the Ferris wheel in the park are popular with children)	32.8	9 (Park Square)
Jiefang Park	30°36′34.6″N, 114°17′43.4″E (WGS84)	Comprehensive (Jiefang Park is a park themed to commemorate the Communist Party of China's leadership in achieving national liberation and often hosts various cultural events. The Jiefang Tower in the park is one of the landmarks of Wuhan and attracts a large number of citizens and tourists)	46	6 (Park Square)
Wuhan Zoo	30°32′23.0″N, 114°14′48.0″E (WGS84)	Zoo (Wuhan Zoo is a modern zoo with animal exhibition and conservation as its main functions, and is one of the most famous tourist attractions in Wuhan)	64.66	8 (Zoological and Botanical Gardens)
Yuehu Park	30°33′11.1″N, 114°15′46.0″E (WGS84)	Theme Park (Yuehu Park is centered on the scenic Yuehu Lake, with the Qintai Grand Theatre as its landmark, which often hosts various performances)	106	21 (Nature and Landscape)
Guishan Park	30°33′21.0″N, 114°16′30.6″E (WGS84)	Comprehensive (The main attraction of Guishan Park is Guishan Mountain, one of the three famous mountains in Wuhan, on which the famous Guishan TV Tower is located. It is one of the tourist attractions in Wuhan)	35.33	14 (Nature and Landscape)
Shahu Park	30°34′25.9″N, 114°20′59.7″E (WGS84)	Comprehensive (Shahu Park is one of Wuhan's famous ecological parks, with functions such as ecological protection, science education, recreation and entertainment)	377	7 (Park Square)

The ranking of each park is not based on a uniform criterion, as they are in different lists in Dazhong Dianping website, with their corresponding list categories in brackets.

### Automated timed data collection and pre-processing

This study used Dazhong Dianping website (https://www.dianping.com/) as a data source to collect data of users’ comments on the seven urban parks. The Dazhong Dianping website is a well-known service rating website for restaurants and tourism in China, which provides large amounts of user ratings and comments on businesses and scenic spots^25^. To obtain sufficient comment data, this study employed a crawler program to automate data collection from the Dazhong Dianping website. The specific steps are as follows:

1. Determination of the scope of data collection. This study selected seven urban parks as the research subjects and determined their scope and keywords. On the Dazhong Dianping website, a search was conducted using the park names as keywords to filter out comment information related to the parks, including park names, comment contents, and comment time. Finally, 26,195 historical comment data from January 2018 to May 2023 were collected to ensure the timeliness and comprehensiveness of the data.
2. Writing of a crawler. This study used Python Requests and the Selenium library to write a crawler for crawling the park comment data from the Dazhong Dianping website by simulating the search and click of users. First, all historical data were crawled, and then a timed task was set up for data crawling once a day in the early morning to obtain real-time new comment data. The updated data were stored in the database for subsequent analysis.
3. Data cleaning and pre-processing: The collected data went through a series of cleaning and pre-processing steps to ensure the accuracy and reliability of the subsequent analysis.
  Processing of duplicate data: A de-duplication operation was performed on all collected comment data to ensure that each comment is unique. Using Python's pandas library, duplicate comment entries were identified and removed based on the combination of comment content and comment time.
  Missing value handling: This process involved removing data rows where the comment content is empty, as these rows did not contribute to the subsequent text analysis.
  Removal of Special Symbols and Non-Textual Content: In addition to Chinese content, the collected comments often contained special symbols (e.g., @, #, &, *) as well as English characters and other non-textual elements. In order to maintain the purity of the comments and improve the quality of analysis, we used regular expressions to match and remove special symbols. Additionally, we utilized the API provided by Baidu Translate to convert the common English expressions into their corresponding Chinese equivalents, ensuring the integrity and consistency of the Chinese text in the comments.
  Removal of emoticons and HTML tags: Comments might have contained emoticons such as “[sun]” or HTML tags. These elements could have interfered with subsequent text analysis. To improve analysis accuracy, we used regular expressions to match and remove all HTML tags, such as “<…> ” and “[…]”, from the comment data. This ensures that the remaining text contains only valuable information for analysis.

After completing the above pre-processing operations, a total of 26,074 valid comments were obtained. These processed data provided a solid foundation for subsequent real-time analysis and processing based on deep learning.

### Training of the deep learning text multi-classification model

In this study, park management was classified into five aspects, including service, facilities, environment, activities, and safety, with reference to the Wuhan City Urban Park Management Regulations, which could provide the basis for comment classification^26^. The Baidu EasyDL platform (https://ai.baidu.com/easydl/) was used to train a text-based multi-classification model to classify the comment data into the above five aspects related to park management and one invalid category (comment data irrelevant to park management and error or irregular data).

Choosing the suitable model is crucial for improving efficiency, as the process of training deep learning models involves several complex steps. The first step is data preprocessing, in which raw data must be cleaned, normalized, and transformed into a suitable format for the model^27,28^. This is followed by the initialization of the model architecture, which determines the layers and parameters of the model^29^. The model then learns iteratively on the training data, continuously adjusting the weights through optimization algorithms such as gradient descent to minimize the error between predicted output and actual labels. To ensure that the model not only performs well on the training set, but also generalizes well to unknown data, a validation set is used to evaluate the model performance. Based on this feedback, the model parameters are further adjusted to achieve optimal performance^30^. After completing model training, deploying the model is also essential, which can be a resource-intensive and time-consuming process.

In the field of text categorization, there have been models such as CNN, LSTM, and BERT that have been widely studied and applied^31^. CNN in text categorization is mainly used to capture the local features of the text through a convolutional layer, which extracts different levels of textual features using multiple convolutional kernels, which can be regarded as filters for learning different patterns in the text. When processing text, CNNs operate on sequences of word embeddings through sliding windows to capture local dependencies^32^. LSTM, as a special kind of recurrent neural network (RNN), is designed to solve the problem of gradient vanishing in traditional RNNs when processing long sequential data. It controls the flow of information by introducing gating mechanisms, such as forgetting gates, input gates, and output gates, to efficiently capture long-distance dependencies^33^. BERT is a Transformer-based model that innovatively captures the deep semantic relationships of large-scale textual data through pre-training and then fine-tuning them for specific tasks. BERT's core is its bidirectional structure, which allows it to simultaneously consider the left and right contexts of each word in the text, resulting in a richer semantic representation^31,34^. Whether it is local feature learning in CNN, long-term dependency capture in LSTM, or contextually relevant representation in BERT, these models are able to improve their performance in tasks such as classification and prediction by learning the intrinsic features of textual data.

Although CNN, LSTM, and BERT are all used in text data processing, they are applied in different scenarios. CNN is particularly suitable for short texts or text classification tasks that require local semantic analysis, such as sentiment analysis or topic classification, due to its expertise in capturing local features. LSTM is suitable for text categorization tasks that require understanding of long-term dependencies, such as sentiment analysis or understanding of complex sentence structures in long texts^33^. In addition, BERT excels in complex text categorization tasks that require deep semantic understanding, especially in context understanding and polysemous word processing, such as question and answer systems or linguistic reasoning^34^. However, each model has its own limitations and cannot meet the diverse needs of all text processing. For example, CNNs have limited performance in processing long texts and understanding complex contexts, while LSTMs, although capable of handling long-distance dependencies, are deficient in computational efficiency and contextual understanding^35^. Meanwhile, although BERT performs well in context understanding and deep semantic analysis, its pre-training and fine-tuning process is resource intensive and its excessive complexity in processing short texts limits its application scope^31^. Therefore, in order to achieve the best performance in different text processing tasks, it is particularly important to choose a platform that can combine the advantages of these models.

For these considerations, the Baidu EasyDL platform became our primary choice for several reasons. Firstly, the Baidu EasyDL platform provides a comprehensive and efficient solution for text categorization and sentiment analysis by integrating a variety of leading deep learning models, such as CNN, LSTM, and BERT. By harnessing the strengths of these diverse models and addressing their individual limitations, it ensures efficient processing and accurate categorization of various types of text data. Secondly, the EasyDL platform provides us with an end-to-end solution, from data preprocessing to model training, optimization, and deployment. Its automated tools greatly simplify the complexity of model development, allowing us to focus on the research problem instead of technical implementation details. This also reduced our reliance on local computing resources. Additionally, EasyDL is built on Baidu's self-developed PaddlePaddle deep learning framework and Kunlun AI chip, ensuring fast and stable model training. PaddlePaddle, as an open-source framework, has extensive community support. This means that problems encountered during model training can be resolved quickly. Furthermore, the Kunlun AI chip offers powerful computing capabilities, making model training faster and accelerating the process of finding the optimal model configuration. The specific steps are as follows:

(1) Data processing. First, 70% of the data was randomly selected from the dataset as the training set and 30% was used as the test set. Considering that this allocation ensures that the model has enough data to learn various patterns during training and provides sufficient samples for validation and testing of the model, we have decided to use this ratio for training. The comment texts in the dataset were uploaded to the EasyDL platform to create a multi-label text classification dataset (see Supplementary Table S1), and the training samples were manually annotated online.

(2) Model training. Given the characteristics of the sample data and the desired classification outcome, we chose a high-precision algorithm with greater accuracy. With EasyDL's high-performance algorithm the training time is significantly reduced for the same amount of training data. The model’s prediction speed is fast, but its accuracy is, on average, 2–5% lower than that of the high-precision algorithm. However, when the training sample size is less than 50 items, using the small-sample training mode can improve accuracy by 1–3% compared with the high-precision model. Overall, high-precision algorithms perform better on larger datasets in terms of accuracy and can improve the precision of the study. The model selection metrics take into account Precision and Recall because, in text categorization, we are not only concerned with the predictive accuracy of the model, but also value its coverage of all positive examples. Our training is performed on the Tesla GPU P40 specification provided by EasyDL, which has 24 GB of video memory, 12-core CPU, and 12 TeraFLOPS of arithmetic power. The training environment of this platform allows us to significantly improve efficiency and shorten the duration of training compared to traditional local environments.

(3) Model calibration. Text multi-classification models are often evaluated with Precision, Recall, and F1 values as core metrics. Precision refers to the proportion of true positive samples correctly predicted by the classifier, and the higher the precision is, the more accurate the model will be. The Recall rate refers to the proportion of samples classified as positive in all the classified samples that are actually positive, and a higher value indicates a wider coverage of the model. The F1 value is a composite metric used to measure the precision and recall of a classification model, and is the summed average of precision and recall. The evaluation metric is calculated as follows^28^:

1 $${\text{Precision}} = \frac{TP}{{TP + FP}}$$

2 $${\text{Recall}} = \frac{TP}{{TP + FN}}$$

3 $${\text{F}}1 = \frac{{2 \times {\text{Precision}} \times {\text{Recall}}}}{{{\text{Precision}} + {\text{Recall}}}} = \frac{2TP}{{2TP + FP + FN}},$$

where *TP*, *FP*, *TN*, and *FN* denote the four outcomes of true positive, false positive, true negative, and false negative for the confusion matrix, respectively^36,37^. A true positive is an outcome correctly predicted as positive by the model. True negative measures the extent to which the model correctly predicts the negative sample. A false positive is an observation that is actually negative, while a false negative is an observation that is actually positive^38^.

The macro-average of a metric is the average across all categories of a given model, and the macro-average of precision, recall, and F1 value serves as an indicator for the overall evaluation of the multi-classification. The formula is as follows^39–41^:

4 $${\text{Macro}}\;{\text{Average}}\;{\text{Precision}} = \frac{{\mathop \sum \nolimits_{i = 1}^{n} Precision_{i} }}{n}$$

5 $${\text{Macro}}\;{\text{Average}}\;{\text{Recall}} = \frac{{\mathop \sum \nolimits_{i = 1}^{n} Recall_{i} }}{n}$$

6 $${\text{Macro}}\;{\text{Average}}\;{\text{F}}1 = \frac{{2 \times \left( {{\text{Macro }}\;{\text{Average}}\;{\text{Precision}} \times {\text{Macro}}\;{\text{Average }}\;{\text{Recall}}} \right)}}{{{\text{Macro}}\;{\text{Average }}\;{\text{Precision}} + {\text{Macro}}\;{\text{Average}}\;{\text{Recall}}}},$$

where *n* denotes the number of classes, ${Precision}_{i}$ indicates the precision value of class *i* ∈ {1, 2, …, *n*}, and ${Recall}_{i}$ represents the recall value of class *i* ∈ {1, 2, …, *n*}. Macro Average Precision represents the average of all category precision rates; Macro Average Recall is the average of all category recall rates; and Macro Average F1 is the harmonic mean of Macro Average Precision and Macro Average Recall^41^.

After four tuning sessions, a well-performing model was successfully trained in this study. The training results for the test set data had a macro-average F1 value of 85.8%, a macro-average precision of 92.7%, and a macro-average recall of 84.3%.

(4) Model deployment. Next, the model was deployed and published on the Baidu Public Cloud to facilitate later call to the API for text classification. Deploying the models on Baidu Public Cloud not only provides fast and stable access, but it also offers significantly better performance than traditional local deployment, especially in highly concurrent scenarios. In addition, Baidu Cloud's built-in security mechanisms provide additional safeguards for the data and models, significantly reducing the risks associated with local deployment.

Using the above methodology, we were able to efficiently and accurately categorize the park management comment data in depth, thus laying a solid foundation of data for sentiment analysis and policy recommendations. Importantly, with the EasyDL platform, we were able to avoid delving into complex modeling techniques and focus more on the methodological aspects of the study. This saved us a significant amount time and resources. As a result, we are confident that this research approach will contribute more effectively to the management strategy of urban parks.

### Data analysis and processing

#### Text classification and sentiment analysis

This step is to call the model API to perform text multi-classification and sentiment analysis on the pre-processed data. The text classification API was used to classify 26,074 historical and real-time data, with a confidence threshold of 0.6, and then the classification results were updated into the data table. In the end, 22,854 comments of different types related to park management were obtained. For sentiment analysis, this study called the deep learning sentiment analysis API of Baidu AI Open Platform to perform sentiment analysis on all the data. For each comment, the sentiment analysis API assigned it with a label of sentiment tendency, including negative (0), neutral (1), and positive (2), as well as the corresponding confidence level (0–1), positive sentiment probability (0–1), and negative sentiment probability (0–1). The sentiment analysis results were then combined with the results of the comment classification for further analysis of the sentiment orientation of different management issues (Table 2).

**Table 2:** Text classification and sentiment analysis results.

Park name	Comment content	Comment time	Text classification	Sentiment tendency	Confidence probability	Positive sentiment probability	Negative sentiment probability
Shahu Park	“Shahu Park is really beautiful this time of year, with a sea of flowers everywhere”	2023-4-22 12:19:00	Environment	2	0.977339	0.989803	0.0101974
Guishan Park	“The parking lot is conveniently located near the west entrance of the park, with tables and benches for resting along the way, and mineral water vending machines”	2022-9-18 16:13:00	Facilities, services	2	0.98064	0.991288	0.00871196
Jiefang Park	“……This time I went and found that there were not many chrysanthemums. There were few Ferris wheel compared to Zhongshan Park, but there was a pigeon house where many adults and children were gathered to watch and feed……Not easy parking nearby”	2020-11-12 22:30:00	Facilities, environment, activities	0	0.905279	0.0426245	0.957376
……	……	……	……	……	……	……	……

The sentiment analysis on park management-related comment data can help further understand users' feelings and needs regarding various issues of park management. This step provides an important basis for subsequent data mining, and also helps park managers and decision makers to better understand the users' sentiment tendencies and needs, so as to adjust park management measures and service in a timely manner, and improve park management and service quality^9^.

The classification and sentiment analysis results of the park comment data can be accessed from the GitHub repository at “https://github.com/SijiaLiu0910/Wuhan-Parks-comment-analysis-data.git”. As it is publicly available and de-identified, its analysis does not constitute human subject research.

#### Quantification of sentiment

In order to quantitatively assess how visitors feel about the park and the performance of the park in terms of different management issues, this study quantified the sentiment of park comments using a formula that considers the positive sentiment probability (*PSP*) and negative sentiment probability (*NSP*) for each comment, which was calculated as follows^42^:

7 $$NS = PSP - NSP,$$

where *NS* denotes the net sentiment score, *PSP* represents the positive sentiment probability, and *NSP* indicates the negative sentiment probability.

The *PSP* was subtracted with the *NSP* for each comment's sentiment score to obtain a continuous range of sentiment values that indicate the sentiment tendency of the comment. This range is usually from − 1 (completely negative) to 1 (completely positive), with 0 indicating neutrality. This calculation makes it possible to obtain a simple and intuitive value to indicate the sentiment tendency of a comment.

By calculating the sentiment score of each comment, the average sentiment score for the park, for each management issues, and for each month can be calculated to assess the park's overall performance, its performance in each management issue and its trends over time using the following formulae:

(1) Average park sentiment score

8 $$GSS = \frac{{\sum \left[ {PSP - NSP} \right]}}{Nv} \times 10,$$

where *GSS* indicates the mean sentiment score of the park, *PSP* represents the positive sentiment probability, *NSP* indicates the negative sentiment probability, and *Nv* indicates the number of valid comments.

(2) Average sentiment scores for each management issue in the park

9 $$TSS_{j} = \frac{{\sum \left[ {PSP_{j} - NSP_{j} } \right]}}{{Nv_{j} }} \times 10,$$

where $TSS_{j}$ denotes the average sentiment score of management issue *j*, $\sum \left[ {PSP_{j} - NSP_{j} } \right]$ stands for the sum of sentiment scores of management issue *j* with comments, and $Nv_{j}$ denotes the number of valid comments of management issue *j*.

(3) Average sentiment score for each month in the park

10 $$MSS_{m} = \frac{{\sum \left[ {PSP_{m} - NSP_{m} } \right]}}{{Nv_{m} }} \times 10,$$

where $MSS_{m}$ denotes the average sentiment score of month *m*, $\sum \left[ {PSP_{m} - NSP_{m} } \right]$ represents the sum of sentiment scores of all comments in month *m*, and $Nv_{m}$ denotes the number of valid comments in month *m*.

It should be noted that only comments with confidence levels greater than or equal to 0.6 are considered as valid, which can ensure that sentiment scores are based on more reliable data, while the average sentiment score of the park was multiplied by 10 to convert the score from a floating-point number within [− 1, 1] to a floating-point number within [− 10, 10] for easier understanding and comparison.

Overall, the sentiment scores can provide a quantitative metric to indicate the degree of positivity or negativity expressed by the visitors. By considering the confidence level of a comment, it is possible to ensure that the sentiment analysis is based on more reliable data, and by using a formula that considers both positive and negative sentiment probabilities and multiplying the score by 10, a more nuanced understanding of the sentiment expression of a comment can be obtained.

#### Feature word extraction

In order to identify the core feature words of each park management problem, the TF-IDF (Term Frequency-Inverse Document Frequency) algorithm was used in this study. TF-IDF is a statistical method that assess the importance of a word in a document. The algorithm is based on a simple principle: words that occur more frequently in a given document and less frequently in other documents tend to have higher importance^43^. Therefore, the TF-IDF algorithm can effectively filter out the characteristic words in each park review and reduce the influence of general and universal words.

The structure of Chinese is different from English, and Chinese does not have clear word boundaries. Therefore, special methods are needed to process Chinese text. We used the Jieba tool for word segmentation processing. Jieba is a word segmentation system designed specifically for Chinese. It can break down sentences into sequences of words with actual meaning^28^. After segmenting the text using Jieba, we further optimized it by using a stop word list specifically designed for Chinese. This list helps filter out frequently occurring words that contribute less to the overall meaning of the text, such as “so,” “general,” and “and.” This step ensures the accuracy of the analysis.

After calculating the TF-IDF values of positive and negative comments for each park, we extracted the top 30 feature words with the highest TF-IDF values and their corresponding number. These feature words with high TF-IDF values reflect key content and sentiment in the comments. They allow us to visualize the specific manifestations of each park's management issues. For example, when negative comments about a park frequently mention words like “dirty” and “crowded,” the high TF-IDF values suggest that the park may have issues with cleanliness and capacity management.

#### Creation of API with Flask and data return

Creation of API with Flask and data return are the final and also critical step in the data processing process. The core goal of this step is to organize the processed data and analysis results into a standardized format and publish them in an accessible way, making them accessible and usable by park managers, as well as ensuring that the data are presented in a dynamic form^44^. Flask provides very simple and easy-to-understand methods for building and running web servers, including creating routes, handling HTTP requests, and returning data^45,46^. In this study, separate routes were created for each of the data queries. For example, there may be one route for returning the average sentiment score of each park and another route for returning sentiment scores of each park over different time periods. The exact creation process and processing logic may vary slightly for each route. However, they generally all receive an HTTP request, then query the database or process the data accordingly based on the content of the request, and finally return the results to the user. When returning data, JSON (JavaScript Object Notation) is a lightweight data interchange format that is easy to read and write, and easy to parse and generate by machine^47,48^.

Overall, by creating and returning data through API with Flask, the data processing process and analysis results can be presented in a visual and easy-to-use way on the web, which allows real-time understanding of the progress and results of the study, and also provides a basis and convenience for future research.

### Visualization of assessment results on the web

For the above processing results, the AntV G2Plot chart visualization tool and the web front-end development languages HTML, CSS, and JavaScript are used to visualize and interact with the charts so that managers can have a more intuitive understanding of the park management situation for decision making. By combining the above processing and visualization methods, this study provides a more comprehensive, accurate, and reliable database and decision support for comprehensive park management assessment.

## Results

### Comprehensive comparison of park sentiment scores

**Figure 2:**
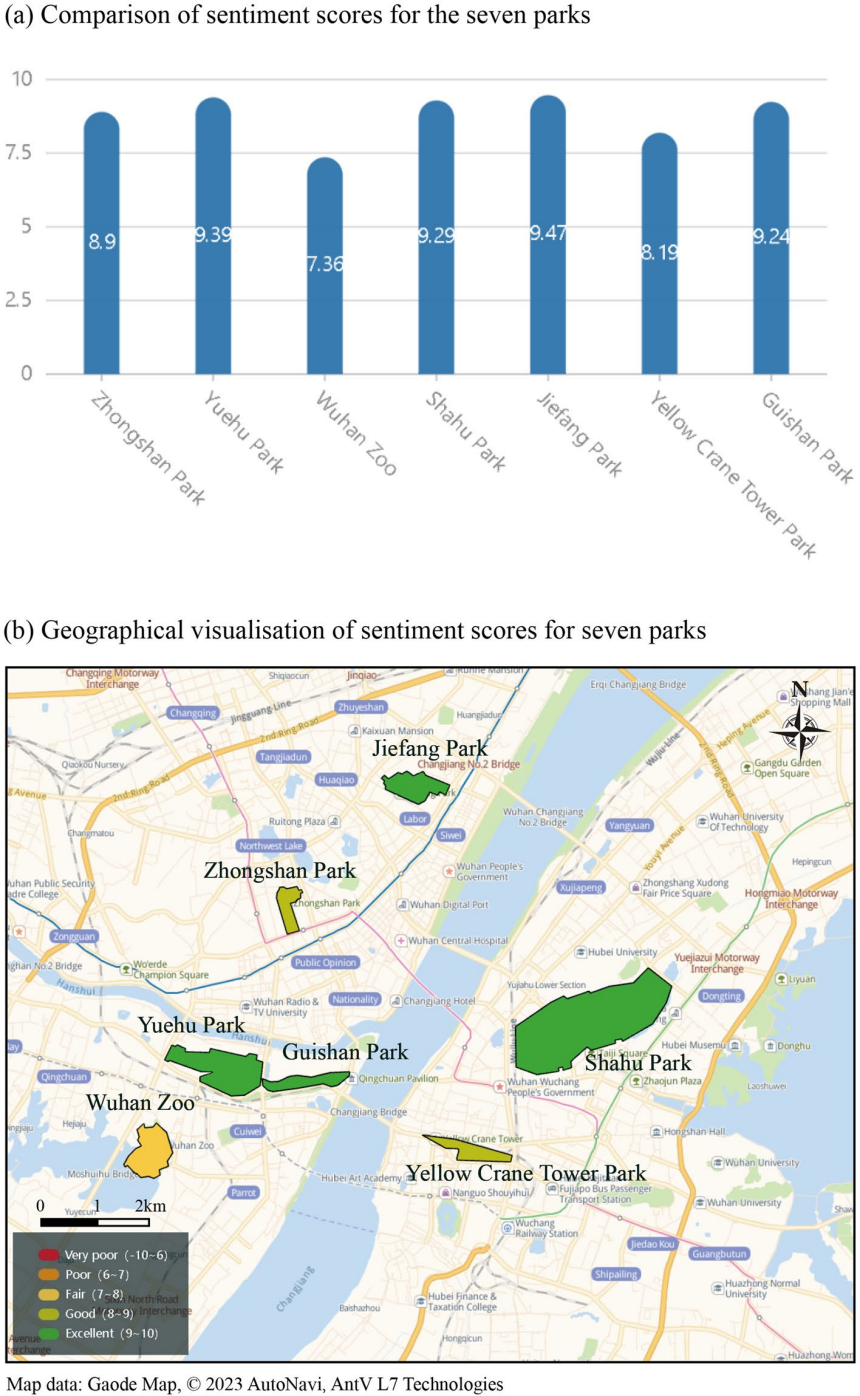
Comparison and geographical visualization of the sentiment scores of the seven parks. Sentiment scores here are calculated as an average of data from January 2018 to May 2023, with sentiment scores changing in real time as the comment data are regularly crawled daily.

This study presents a comprehensive comparison of the sentiment scores of seven parks in Wuhan at a macro level with a bar chart and a geographically classified map (Fig. 2). To display the different sentiment score levels of the parks with different colors on the map, the sentiment scores of the parks were classified into five categories: excellent (9–10), good (8–9), fair (7–8), poor (6–7), and very poor (− 10–6), according to the distribution of the application scenarios and data of this study.

Our results demonstrate that Jiefang Park and Wuhan Zoo have the highest (9.47) and lowest (7.36) sentiment score, respectively. As shown in Fig. 2b, the sentiment scores of Jiefang Park, Shahu Park, Guishan Park, and Yuehu Park are classified into the “excellent” category, while those of Zhongshan Park and Yellow Crane Tower Park are classified into the “good” category, and that of Wuhan Zoo is classified into the “poor” category.

Taken together, the relatively high sentiment scores of Jiefang Park, Shahu Park, Guishan Park, and Yuehu Park indicate that visitors to these parks are more satisfied with their management. In contrast, Zhongshan Park, Yellow Crane Tower Park, and Wuhan Zoo have relatively low sentiment scores and therefore need further improvement of park management quality, particularly Wuhan Zoo.

### Temporal trends in park sentiment scores

**Figure 3:**
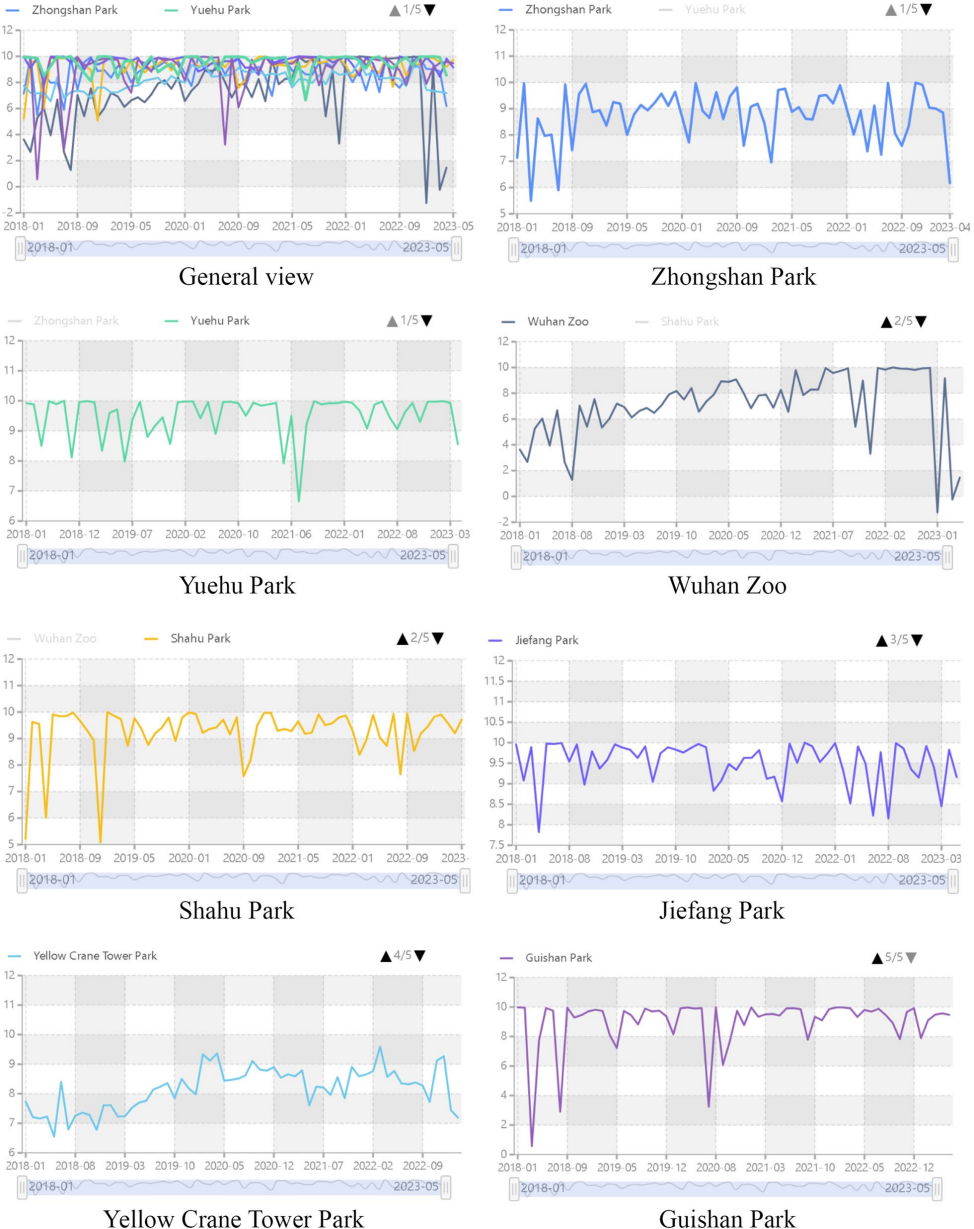
Temporal trends of sentiment scores for the seven parks from January 2018 to May 2023. The overall trend in sentiment scores and details of a particular time period can be observed by sliding the thumbnail below the line graph. The trends of individual sentiment scores for each park can be viewed by clicking the page flip symbol and the small circle next to the legend. When the mouse is hovered over the line, the sentiment score for each park at that time point can be displayed.

Based on the temporal trends in sentiment scores (Fig. 3), the parks can be divided into two categories: parks with a clear upward or downward trend in sentiment scores, including Wuhan Zoo and Yellow Crane Tower Park, and parks with fluctuating sentiment scores, including Yuehu Park, Jiefang Park, Zhongshan Park, Shahu Park, and Guishan Park.

The sentiment score of Wuhan Zoo shows an upward trend until August 2021 and then exhibits fluctuations. Between August 2021 and December 2022, the sentiment score dropped sharply from 9.93 to 1.46. This decline can probably be attributed to the closure of the zoo for renovation in the second half of 2021, which resulted in a decrease in the public comments of the park.

The sentiment scores of some parks show a more pronounced seasonal pattern. There are obvious seasonal fluctuations in sentiment scores of Yellow Crane Tower Park throughout the year, with relatively high and low sentiment scores in spring and summer, respectively. The sentiment score reached historical high of 9.36 and 9.39 in April 2020 and March 2022, respectively, while was fairly low in May (6.55) and July 2018 (6.79) and May 2012 (7.61). This may be due to the tourist season, which leads to more visitors in the park, and poor park management services, which negatively affects the park's score. The sentiment scores of Yuehu Park and Shahu Park are significantly lower in summer and winter, which may be related to the lake management in summer and the waning scenery in winter.

It can also be observed from the charts that most parks experience fluctuations in sentiment scores during specific time periods. These fluctuations can be influenced by a variety of factors, including park management, facility updates, and seasonal changes. These data analyses provide valuable feedback to park managers, helping them adopt appropriate management and service strategies for different time periods.

### Performance of different management issues in the park

**Figure 4:**
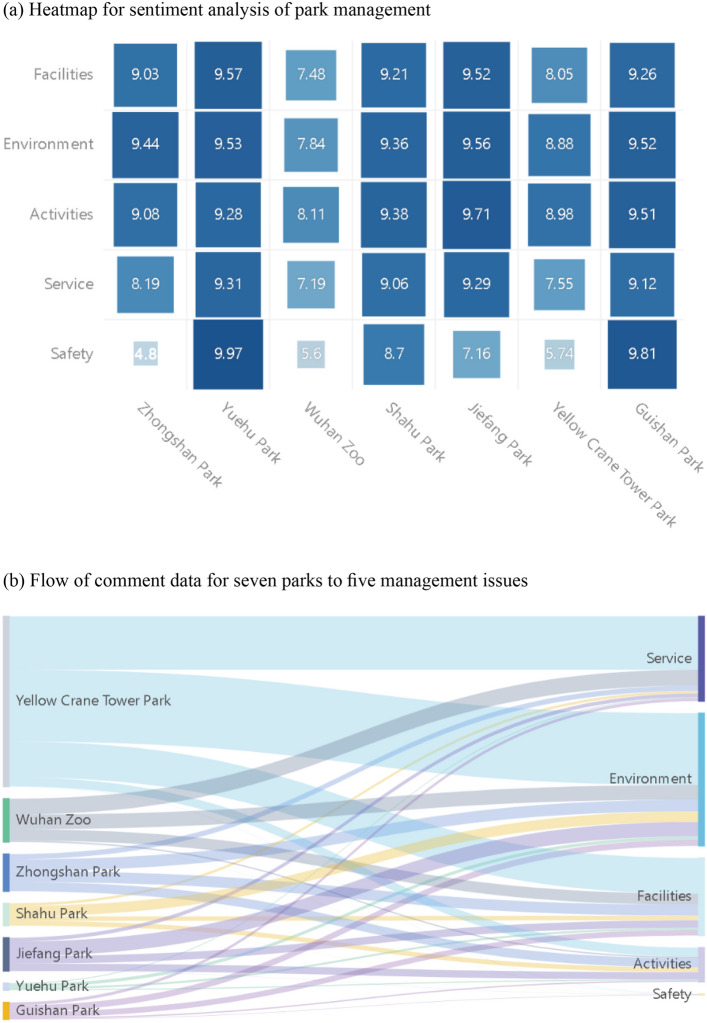
Performance of parks in different management issues. (**a**) The size and color shade of each square of the heatmap represents the intensity of the indicator. The heatmap allows a better understanding of the differences and links between the sentiment scores of different park management issues and thus facilitates decision-making. (**b**) The Sankey diagram consists of a series of directed arrows, the width of which indicates the magnitude of the flow, with the start point indicating the source of outflow and the end point indicating the destination of inflow. The Sankey diagram can reflect visitors' concerns about different management issues in different parks.

In order to understand the performance of different parks in different management issues and the focus of visitors on different park management issues at a meso level, we used heatmaps and Sankey diagrams to present the results of each issue (Fig. 4). First, Yuehu Park and Guishan Park generally have the highest scores among all issues with good performance; in contrast, Wuhan Zoo and Yellow Crane Tower Park generally have lower scores with poorer performance. Secondly, the focus of each park's management differs, with Zhongshan Park showing the highest score in the environment (9.44), Yuehu Park and Guishan Park exhibiting the highest score in safety (9.97 and 9.81), and Wuhan Zoo, Shahu Park, Jiefang Park, and Yellow Crane Tower Park showing the highest scores in activities (8.11, 9.38, 9.71, and 9.89). Among all management issues, visitor satisfaction is low with safety-related issues, particularly Zhongshan Park, which has a sentiment score as low as 4.8 for safety.

Second, the problems concerned by the visitors differ among different parks. For example, about 90% of the comments for Yellow Crane Tower Park and Wuhan Zoo are concerned about service, environment, and facilities, while the remaining 10% of comments are concerned about activities and safety. About 90% of comments for Zhongshan Park are concerned about the environment, facilities, and activities, and approximately 50% of the comments for Shahu Park, Jiefang Park, Yuehu Park, and Guishan Park are concerned about the environment. The smallest amount of visitor comment data is concerned with the safety for all parks, which may indicate that visitors are relatively less concerned with park safety.

### Hotspot mining and prominent problems

**Figure 5:**
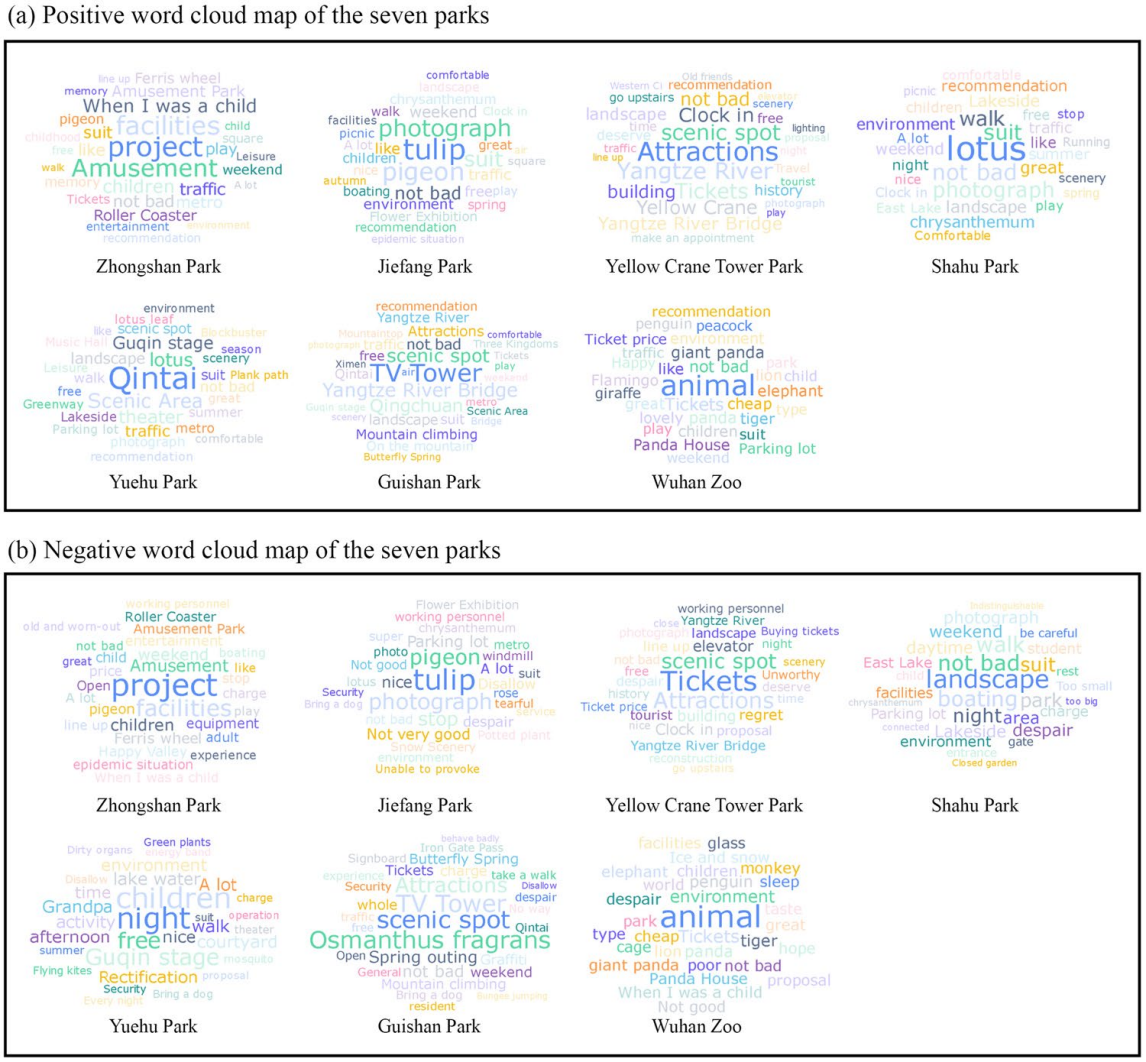
Positive and negative word cloud maps for the seven parks. The size and color shade represent the frequency of the feature words in the comments.

The positive word cloud and negative word cloud maps can reflect the specific hotspots and prominent problems in each park (Fig. 5). The results reveal that each of these parks is unique and attracts different types of visitors. The feature words in Zhongshan Park are mainly related to ferris wheel and recreational activities, such as “project”, “facilities”, “amusement”, “roller coaster”, and “ferris wheel”. In addition, words such as “childhood”, “when I was a child”, and “memory” indicate that Zhongshan Park occupies an important place in people's childhood and memories. Wuhan Zoo is popular with visitors because of its unique animals, with animals such as “panda”, “tiger”, and “flamingo” being the most popular. In terms of admission fees, visitors are satisfied with the "ticket price" of Wuhan Zoo. Yuehu Park, Shahu Park, and Jiefang Park are featured with natural plants, with "lotus" as the special feature of Yuehu Park and Shahu Park, while Jiefang Park are featured with “tulip” and “chrysanthemum”. These parks are popular with visitors due to their beautiful plants and surroundings, facilities for walking, running, and picnicking, and free services. In contrast, Jiefang Park is popular with visitors in terms of activities and facilities. Yellow Crane Park is popular with visitors owing to its scenic spots, and “attractions”, “scenic spot”, “Yangtze River”, “Yellow Crane”, and “building” are more frequent feature words. In contrast, feature words such as “TV tower”, “mountain climbing”, “scenic spot”, and “Qintai” appear more frequently in Guishan Park. These high-frequency feature words indicate that visitors value the experience of climbing and enjoying the architecture and natural beauty of the park.

The negative word cloud map can reveal specific problems in park management, which can assist park management to further improve the quality of park services and visitor experience based on the suggestions and concerns of the visitors. As shown in Fig. 5b, “facilities” and “project” of Zhongshan Park are the hotspots of negative comments from visitors, indicating that the visitors are less satisfied with the quality and services of the park's amusement facilities and infrastructure. Wuhan Zoo receives more negative comments, mainly in the issues of “animal”, “tickets”, and “environment”. In addition, visitors are also concerned about and interested in animal welfare and the sustainable development of the zoo. There are fewer negative comments on Yuehu Park, Shahu Park, Jiefang Park, and Guishan Park. There are some negative comments on the nights in Yuehu Park and Shahu Park, which are possibly related to their nighttime management, while “lake water” and “charge” are specifically mentioned for Yuehu Park, suggesting that more attention should be paid to the lake management in Yuehu Park. Negative comments on the Yellow Crane Tower Park are focused on “tickets”, “building”, “elevator”, and “line up”, indicating that the visitors are concerned about the park's admission prices, service facilities, and operation efficiency.

## Discussion

### Dynamic nature and multiple needs of park management

This study constructs an intelligent assessment framework based on deep learning text mining for in-depth analysis of social media data in park management. Firstly, the data show that parks predominantly with natural scenery generally have higher sentiment scores than those predominantly with facilities and buildings. For example, Yuehu Park, Shahu Park, Jiefang Park, and Guishan Park have higher sentiment scores than Zhongshan Park, Yellow Crane Tower Park, and Wuhan Zoo, indicating that visitors prefer parks with natural scenery as their main feature. Therefore, in park design and management, emphasis should be laid on the protection and enhancement of natural sceneries, and on rational planning and configuration of park facilities and buildings so as to improve the overall satisfaction and attractiveness of parks. There is also a correlation between the sentiment ratings of the parks and the socio-economic characteristics of the areas in which they are located, such as per capita income and education level (see Supplementary Tables S2 and S4). For example, Yellow Crane Tower Park and Zhongshan Park are situated in areas with higher income and education levels, and thus local residents have higher expectations and more stringent evaluations of park services and facilities. This could explain why these parks have relatively lower ratings. On the other hand, Yuehu Park and Guishan Park are located in Hanyang District, which has a relatively lower level of education. As a result, exposure to nature is the most convenient and economical way for visitors there to enjoy their leisure^49^, leading to higher ratings for these parks. It is worth noting that the high population density in the Yellow Crane Tower Park area exposes the park to a greater flow of visitors, thus making park facilities and management more challenging.

Secondly, park sentiment scores are influenced by time of the day, season, and specific events. For example, the sentiment score of Wuhan Zoo decreased during the closure and renovation of the park, and the sentiment score of Yellow Crane Tower Park showed a downward trend during the peak summer visitor season. These findings clearly indicate that park management should be a process of dynamic adjustment according to the actual situation rather than a static and unchanging strategy^3^. Targeted adjustments on management strategies, such as improving the quality of park services during peak tourism periods or giving visitors advance notice during renovation, may help to reduce negative public perceptions and improve the overall sentiment score.

In addition, the differences in satisfaction ratings of individual parks across different management aspects reveal the diverse public demand for parks. For example, Zhongshan Park received high environmental ratings, which may be attributed to its proximity to numerous shopping centers, restaurants, and business centers. Zhongshan Park’s presence in the center of the city creates an oasis, providing a peaceful and relaxing environment for people, especially patients in the surrounding hospitals. This enhances its ratings in terms of environment. Yuehu Park and Guishan Park, on the other hand, scored high on safety management. This can be attributed not only to the park's own good management practices but also to the presence of its neighboring residential areas, which creates a safer living environment. This, in turn, influences visitors' perception of safety. As for the Wuhan Zoo, its high activity ratings may be attributed its surrounding schools and neighborhoods, which attract numerous families and student visitors to participate in various activities. This further implies that each park has its own unique management priorities and challenges and is closely related to the surrounding user population. For example, although Zhongshan Park was highly rated in terms of environment, it scored the lowest in terms of safety. This is because the park is located in a busy commercial area and is close to schools and hospitals, which attracts a large number of students and patients. These groups are more sensitive to safety concerns. To address safety concerns, managers should regularly inspect all facilities to ensure they meet safety standards, and install additional warning signs and fences to guide and protect the public. Meanwhile, first aid equipment and professional first aiders should be available in the park to deal with emergencies. Therefore, managers should consider the characteristics and needs of the surrounding user groups when developing park management strategies.

Finally, our results also reveal the characteristics of each park and its unique management problems. For example, Zhongshan Park is featured with amusement facilities, but the quality and service of the facilities and programs receive rather low scores. This may be related to the fact that the population of the area includes many young people and schoolchildren, who have higher expectations of play facilities and services. The natural environment of Yuehu Park is highly appreciated by visitors, but negative terms appear in the evaluation of nighttime management and lake treatment. Considering that the park site has many young families who are likely to be more active at night, it becomes especially critical to strengthen nighttime management. Based on these analyses, park managers should develop management strategies tailored to the characteristics and audiences of each park. Parks focusing on natural beauty could strengthen environmental protection measures, while facility-oriented parks should improve their quality and service levels, especially considering the age composition of the population in the area (see Supplementary Table S3).

### Common challenges and solution strategies for park managers and urban planners

Based on the above findings, the assessment framework of this study not only identifies potential issues and challenges in the current management strategies for park managers, but also offers guidance for local officials and planners to better address the needs and expectations of the public.

For park managers, their priority should be to focus on the quality of facilities and services in their parks. Considering the socioeconomic characteristics of individual park locations, such as per capita income and educational attainment, managers should adjust their service standards to meet the expectations of visitors from different economic and educational backgrounds. In addition, considering the population density and visitor flow of the parks, managers should also enhance the safety management of the parks, especially at night, to ensure the safety of the visitors.

As for local officials and planners, they need to plan and make decisions at a more macro level. For example, given the relationship between the park and the surrounding community, officials and planners should strengthen their interaction and communication with community residents to understand their needs and feedback, and formulate long-term plans for the park accordingly. In addition, officials and planners should also consider the positioning and features of the parks to ensure that they can provide the greatest value to the local community, taking into account the age, income, and education distribution of the local residents.

Finally, because park management and urban planning are inextricably linked, cooperation between park managers, local officials, and planners is especially crucial. They need to collaborate in order to integrate resources and information, ensuring that park management strategies and city master plans are aligned to provide a superior park experience for the public and to promote sustainable urban development.

### Urban park management and intelligent assessment

**Figure 6:**
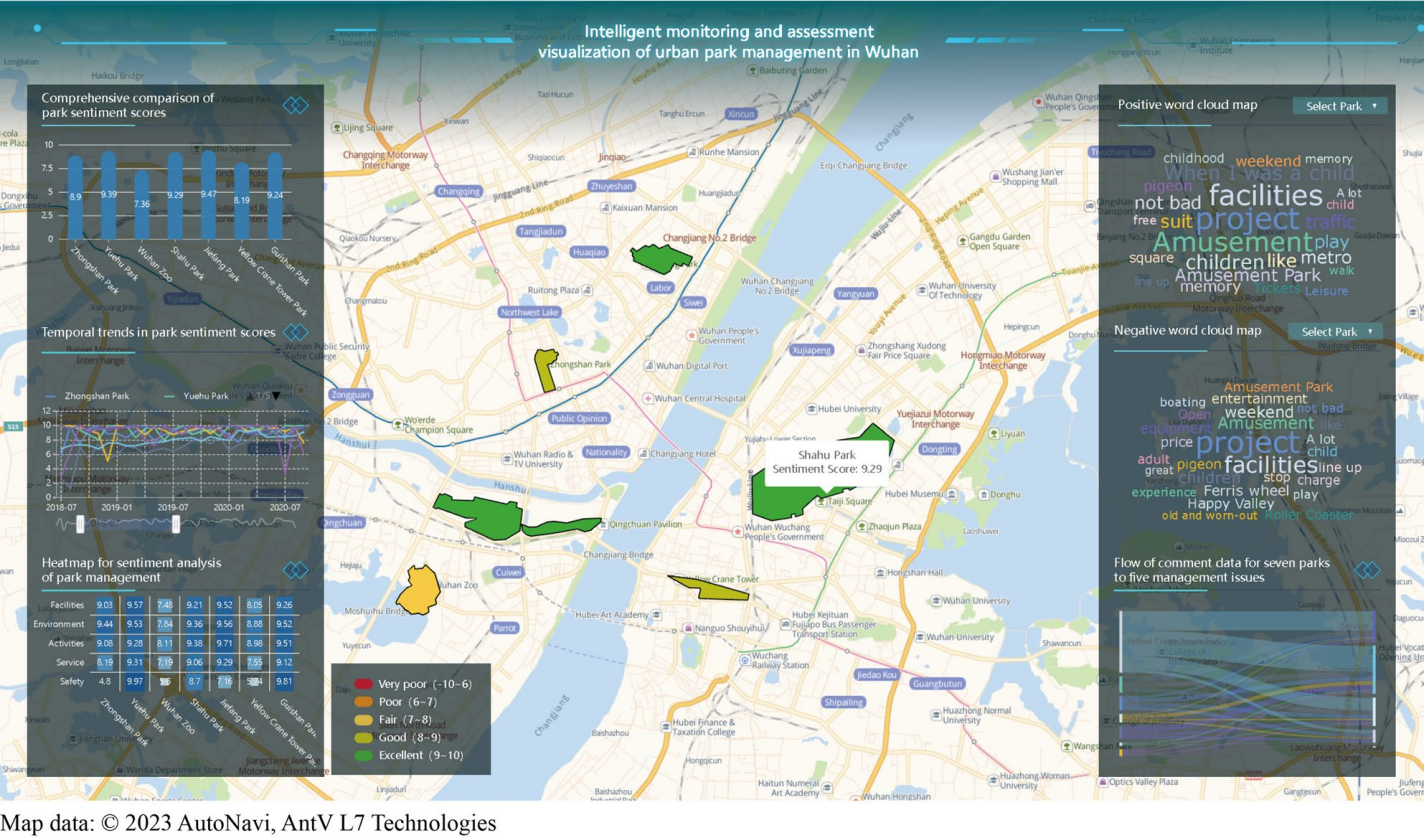
Visualization of management assessment of urban parks in Wuhan. As shown in the animation (Supplementary Animation S1), we have created a website for the project that supports real-time monitoring to assess visitor satisfaction on different park management issues, which provides visualization and dynamic interaction of multiple data analysis results, and can be used as part of a smart park management platform to provide decision support to park managers.

The assessment framework of park management proposed in this study employs users’ comments on social media for real-time analysis, and is characterized by intelligent assessment and problem extraction functions. The framework classifies users’ comments into multiple categories such as facilities, safety, environment, activities and services through a deep learning text multi-categorization model, based on which quantitative sentiment scores can be obtained and prominent park problems can be found. The progressively refined analysis from macro to micro level can help park managers to comprehensively understand the current state of park management from different perspectives and target improvements to the problems (Fig. 6).

First, compared to conventional park management assessment methods such as questionnaires, on-site observations, and expert assessments^3–6^, the present framework can automate the processing of a large amount of user review data. This automation saves a great deal of time and human resources. For example, a newly built park often attracts many visitors to experience it and share their feelings on social media platforms. However, using the traditional approach may take weeks to fully summarize all these feedbacks. With the framework, it only takes a few hours to consolidate and analyze these comments. This allows for the quick identification of key issues and areas for improvement, helping the park to upgrade and renovate more accurately.

Second, the framework is not limited to a specific type of park or urban environment but can be applied to parks of all sizes and geographic locations. Especially when parks are located in different areas, managers do not need to conduct offline research in each place. Instead, they can use the framework to synchronize and analyze the data of the parks in different locations, which provides a more convenient macro-guidance method for managers and planners.

Most importantly, through feature word extraction, this framework can be more targeted to find and solve the main problems in park management. For example, when a large number of users mention “safety issues” or “aging facilities” in their comments, the manager can quickly identify these issues, and then improve and maintain them in a more focused manner.

Furthermore, the framework can be adjusted and optimized according to different types of green space, offering a considerable level of scalability and flexibility. For instance, in different spatial contexts such as national parks and nature reserves, the dimensions of text categorization can be customizes based on the specific management characteristics of each location. This approach enables more precise and in-depth assessments for different areas. In conclusion, the intelligent assessment framework proposed in this study provides a new, efficient and professional tool for park managers and urban planners, aiming to better meet the public's needs and improve the service level and management efficiency of parks.

### Limitations and future research

Although the assessment framework of park management proposed in this study brings a unique perspective and novel approach for park management, the framework still has some limitations in its implementation. Firstly, while social media data can provide a wealth of user feedback and sentiment information for park management, the coverage of this information source is not comprehensive enough. For example, the feedback from some older or less accustomed park visitors may be overlooked. Secondly, the text classification model based on deep learning in this study may not be applicable to the comprehensive assessment of all types of park management. Therefore, more targeted models can be built for specific parks to better represent park management problems and help managers to more accurately improve park service quality and visitor experience. In addition, the methodology of the present study is focused on intelligent assessment of park management, while further research on early warning and application of AI is needed in the future.

In summary, the limitations and challenges of this study offer a variety of ideas and approaches for future research. Firstly, in order to gain a more comprehensive understanding of park use, more data sources can be integrated in the future, including but not limited to questionnaire data and park equipment use data. Secondly, in the future, other machine learning techniques or a combination of methods can be considered to create different classification indicators to better respond to different types of park management problems. In addition, future research can develop an early warning mechanism to predict possible park management problems based on real-time feedback from users and provide timely warnings to park managers. Finally, by incorporating AI technology, future research can provide park managers with more targeted management advice to more effectively solve management problems.

## Conclusion

Parks are an important part of the urban public environment, and their quality of management directly affects the quality of urban life. The development of smart cities also has an urgent need for information technology to optimize park management^50^. This study applies deep learning techniques to build the first intelligent assessment framework for park management based on user comments in social media. The framework provides a comprehensive, real-time understanding of the operational status of urban parks in terms of facilities, safety, environment, activities, and services, and accurately detects the major management problems to help park managers better carry out facility construction, activity planning, and environmental management. This framework not only shows the ability to improve the efficiency of park management and solve problems in a timely manner in the experiment, but also has certain extension application value, which can provide reference for other environmental management, such as scenic tourism areas, wetland parks, and national parks^51–54^. Future work can further enhance the scale, accuracy, early warning capability, and intelligence of the assessment framework to meet the increasingly changing needs of urban park management. Overall, this study pioneers a new paradigm for park management based on big data and AI, offering new possibilities for park management in smart cities.

### Supplementary Information

Supplementary Video 1.

Supplementary Information.

## Data Availability

The datasets generated during and/or analysed during the current study are available in the GitHub repository, https://github.com/SijiaLiu0910/Wuhan-Parks-comment-analysis-data.git.
