# Supplementary material for: A new framework for assessment of park management in smart cities: a study based on social media data and deep learning
Source: Sci Rep. 2024 Feb 13;14:3630. doi: 10.1038/s41598-024-53345-0 (PMC10864378; doi:10.1038/s41598-024-53345-0)
Supplement: Supplementary file 2 — Supplementary Information. [file 41598_2024_53345_MOESM2_ESM.pdf]

Supplementary Information

A new framework for assessment of park management in smart cities: a study based on social media data and deep learning

Sijia Liu[1],Chuandong Tan[1],Feiyang Deng[2], Wei Zhang[1] ,Xuefei Wu[1,✉]

[1] College of Horticulture and Forestry, Huazhong Agricultural University, Wuhan 430070, China
[2] College of Urban Design, Wuhan University, Wuhan 430072, China
[✉] email: wuxf@mail.hzau.edu.cn

Table S1 Manually annotated text classification dataset

Text content	Text type
"Overall not bad, good for taking kids and safe. "	Safety
"We went to see the annual tulip exhibition in Jiefang Park a couple of months ago. The tulips were really pretty, and this year the colors were even more beautiful, with a nice mix of pink and white. The plum blossoms were also in bloom. We recommend going to see the tulip exhibition every time when there is one in Jiefang Park, it's worth it. "	Activities, Environment
"The bad comment is for the project fee, not for the Yellow Crane Tower, though it looks good. But if I have to pay to go in and take a picture, don't even think about it. I'm not a big ingrate. You guys go in and help me see it. I won't go. "	Services
"A memory from my childhood, and now I can go with my children again when I have them. The facilities are much bigger and better than before, and I hope that barrier-free access will be added in front of the venue to make it easier for wheelchairs and prams to get in and out. "	Facilities, services, environment
"The zoo has been repaired than before. A lot of protective nets have been repaired. There are still more animals. They are all so cute. The weather was particularly good when I went that day. The animals were out and there were a lot of people, but the smell of the zoo is still bad. "	Safety, facilities, environment
"Very good for my first visit, very impressive. "	Invalid
"I had the opportunity to visit Wuhan for a few weeks on business and made a detailed tour of the sights here. A great place to relax. "	Invalid
......	......

Table S2 Per capita income and resident population in the regions where the seven parks are located

Region	Per capita disposable income of urban permanent residents (yuan)						Number of resident population (10,000)				
	2018	2019	2020	2021	2022	Average value	2018	2019	2020	2021	Average value
Jiangan district	52510	57503	37382	63709	67595	55739.8	96.27	96.28	96.53	105.1	98.545
Jianghan district	52917	57579	56090	62318	66555	59091.8	72.97	72.98	64.79	68.9	69.91
Hanyang district	47862	52025	51457	56108	59220	53334.4	66.24	67	83.73	90	76.7425
Wuchang district	52583	57501	57109	63258	67180	59526.2	128.28	128.54	110.22	127	123.51

Note: Jiefang Park is located in Jiangan District; Zhongshan Park is located in Jianghan District; Yuehu Park, Guishan Park and Wuhan Zoo are located in Hanyang District; Yellow Crane Tower Park and Shahu Park are located in Wuchang District. Data from Wuhan Municipal Bureau of Statistics(https://tjj.wuhan.gov.cn/).

Table S3 Age composition of the population in the region where the seven parks are located (2020)

Region	Share of resident population (%)			
	0-14 years old	15-59 years old	60 years and over	
				Of which: 65 years and over
Jiangan district	12.9	64.57	22.53	15.21
Jianghan district	11.54	68.71	19.75	13.14
Hanyang district	13.44	67.43	19.14	12.33
Wuchang district	11.58	67.5	20.92	14.64

Note: Data from Bulletin of the Seventh National Population Census of Wuhan (https://tjj.wuhan.gov.cn/ztzl_49/pczl/202109/t20210916_1779157.shtml).

Table S4 Number of persons with various types of educational attainment per 100,000 population in the regions where the seven parks are located (persons/100,000)

Region	College	High school	Junior high school	Primary school
Jiangan district	34858	24651	23282	10903
Jianghan district	33040	24656	25773	10781
Hanyang district	31998	23603	25613	11335
Wuchang district	45444	21528	18408	9194

Note: Data from Bulletin of the Seventh National Population Census of Wuhan (https://tjj.wuhan.gov.cn/ztzl_49/pczl/202109/t20210916_1779157.shtml).

Animation S1 Visualization of management assessment of urban parks in Wuhan

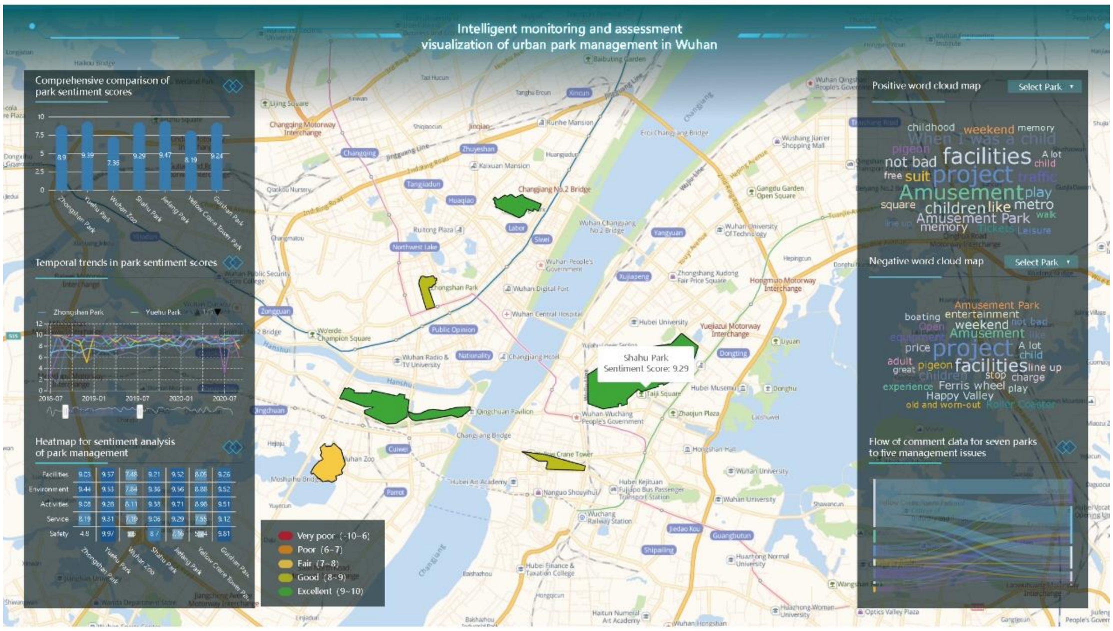

Map data: © 2023 AutoNavi, AntV L7 Technologies

Note: As shown in the animation (Supplementary Animation S1), we have created a website for the project that supports real-time monitoring, aiming to assess visitor satisfaction with various park management issues. The site prominently features a central map displaying key data, while charts presenting additional analysis results flank both sides. Users can intuitively navigate by zooming and panning the map, sliding the timeline, and toggling selection bars to view sentiment scores for each park, temporal trends, and highlight issues and hotspots specific to different parks. In summary, the website provides visualization and dynamic interaction of multiple data analysis results, and can be used as part of a smart park management platform to provide decision support to park managers.
